# The Impact of Oxytocin on Stimulus Discrimination of Zebrafish Albino and Non-Albino Models

**DOI:** 10.3390/ijms26052070

**Published:** 2025-02-27

**Authors:** Ana-Maria Danila, Alexandra Savuca, Alin Stelian Ciobica, Irina Luciana Gurzu, Mircea Nicusor Nicoara, Bogdan Gurzu

**Affiliations:** 1 Department of Biology, Faculty of Biology, Alexandru Ioan Cuza University of Iasi, Bd. Carol I No. 20A, 700505 Iasi, Romania; danilamariuca@yahoo.com (A.-M.D.); alin.ciobica@uaic.ro (A.S.C.); mirmag@uaic.ro (M.N.N.); 2 Centre of Biomedical Research, Romanian Academy, Bd. Carol I, No. 8, 700506 Iasi, Romania; 3 Academy of Romanian Scientists, Str. Splaiul Independentei No. 54, Sector 5, 050094 Bucharest, Romania; 4 “Ion Haulica” Institute, Apollonia University, Păcurari Street 11, 700511 Iasi, Romania; 5 Department of Preventive Medicine and Interdisciplinarity, Faculty of Medicine, “Grigore T. Popa” University of Medicine and Pharmacy, 700115 Iasi, Romania; irina-luciana.gurzu@umfiasi.ro; 6 Department of Morfofunctional Sciences, Faculty of Medicine, “Grigore T. Popa” University of Medicine and Pharmacy, 16th Universitatii Street, 700115 Iasi, Romania; bgurzu@yahoo.com

**Keywords:** social discrimination, albino, oxytocin, zebrafish, predator

## Abstract

Zebrafish have the ability, to a certain extent, to distinguish between different types of stimuli, including distinguishing between videos of conspecifics and non-conspecifics, a skill known as stimulus discrimination. In this study, we investigated the effects of oxytocin on this ability in albino and non-albino zebrafish models, focusing on the correlations between albinism, sensory deficiencies, and socio-emotional behaviors. Our hypothesis is based on the premise that oxytocin influences socio-emotional behaviors in zebrafish, with varying effects depending on phenotype (albino vs. non-albino), social context, and treatment duration. Studies have shown that albino zebrafish have more pronounced sensory deficiencies, meaning they may benefit more from oxytocin in terms of increased social comfort and interactions with conspecifics, while non-albino zebrafish would experience a reduction in defensive behaviors and anxiety. To test this, two experiments were conducted: one assessing the responses to video predator stimuli and the other comparing social interactions with real and video conspecifics. The results showed significant differences between the two groups: non-albino zebrafish exhibited stronger long-term reductions in anxiety-related behaviors, such as reaction speed and freezing, suggesting that oxytocin regulates defensive responses and aggression. Meanwhile, albino zebrafish showed greater improvements in social interactions, reflecting the nuanced, phenotype-dependent effects of oxytocin. These results not only confirm existing research but also highlight the therapeutic potential of oxytocin in treating socio-emotional deficiencies.

## 1. Introduction

Albinism is an autosomal recessive disorder characterized by reduced melanin biosynthesis in melanocytes in the epidermis and hair follicles; the absence or reduction in pigmentation can occur in the skin, hair, and eyes, known as oculocutaneous albinism, or it can affect only the pigmentation of the eyes, known as ocular albinism [1,2]. The World Health Organization (WHO) recognizes albinism as a genetic condition that affects individuals from all social classes and from all countries around the world [3]. The global incidence of albinism is 1 in 17,000–20,000 people in Europe and the United States but higher among populations in Africa (1 in 1000), particularly within the Tonga tribe in Zimbabwe, an isolated rural community [4]. Consanguinity and limited geographical mobility are relevant factors in assessing the current and future prevalence of albinism [5]. There are also much rarer forms of albinism, such as Hermansky–Pudlak syndrome and Chediak–Higashi syndrome, characterized by more severe phenotypes that affect a wide range of cell types beyond the pigmentary cells. Studies indicate that albino individuals in sub-Saharan Africa have approximately 1000 times higher risk of developing skin cancer compared to the general population, being extremely vulnerable to the harmful effects of ultraviolet (UV) radiation. This increased susceptibility significantly contributes to the premature mortality of individuals with albinism, with many dying from skin cancer before the age of 30 and, frequently, between the ages of 30 and 40 [6,7,8]. Albinism human patients often suffer from reduced visual acuity, refractive errors, translucent iris, nystagmus, foveal hypoplasia, hypopigmentation of the fundus, and abnormal decussation of the optic nerve fibers at the level of the optic chiasm, which can lead to strabismus and deterioration of stereoscopic vision [9]. Recently, new genes associated with albinism have been discovered, both in oculocutaneous and syndromic forms. Specifically, two new genes associated with oculocutaneous albinism (*OCA*) have been identified: *SLC24A5* and *C10orf11*, referred to as *OCA6* and *OCA7*, respectively. These discoveries contribute to understanding the genetic complexity of albinism and may facilitate more accurate diagnosis and management of this condition [10,11]. Thus, albinism and its numerous forms have been extensively investigated in genetic association studies [12], but qualitative studies regarding the life experiences of individuals with albinism show that, aside from medical concerns, individuals with albinism also face psychological and social challenges, such as depression, anxiety, social discrimination, abuse, and stigmatization affecting self-esteem and educational and professional opportunities [13]. Although albinism affects a small number of people compared to other major issues, its extensive implications make it a public health problem that requires greater attention, especially for increasing awareness and knowledge in the field [14,15,16].

Oxytocin, produced in the paraventricular nuclei of the hypothalamus, regulates social behaviors in animals and promotes prosocial behaviors by encouraging closeness and reducing avoidance [17,18]. In humans, social bonds are often reflected through emotional experiences towards others. Therefore, the effects of oxytocin in humans should influence behavior and our emotional perception of others [19]. Regarding the connections between oxytocin and affective disorders, it is important to note that oxytocin acts as a modulator of the neuroendocrine axis, specifically regulating cortisol levels, especially under stress conditions [20]. Oxytocin is crucial in the mesocorticolimbic dopaminergic circuit (reward system), with extensive implications in addictive behaviors, motivation, survival, reproduction, feeding, drinking, and sexuality [21]. These processes are significantly disrupted in depression, manifested by decreased motivation, reduced appetite, low libido, and suicidal behavior. At the same time, some clinical studies have clearly indicated the involvement of oxytocin in other psychiatric disorders and the possible beneficial effects of oxytocin administration in depression and anxiety [22,23].

Oxytocin is a neuropeptide synthesized in the hypothalamus and released by the pituitary gland, playing an essential role in regulating social behavior, emotional responses, and stress levels. Olff et al. (2013) suggest that oxytocin plays a significant role in the regulation of social behavior, emotional responses, and stress, highlighting its influence on brain regions associated with these processes, such as the amygdala and prefrontal cortex [24]. For instance, Parker et al. (2019) suggest that oxytocin interacts with the amygdala and dopaminergic system to mediate social rewards. This aligns with the findings of our study, where we observed that albino zebrafish showed an increased preference for social interactions after oxytocin treatment, indicating a more intense activation of the reward system [25]. It is the mechanism of action that involves the activation of oxytocin receptors in various brain regions, including the amygdala and prefrontal cortex, structures involved in processing emotions and social interactions. Studies have shown that oxytocin modulates stress responses by regulating the hypothalamic–pituitary–adrenal axis, reducing cortisol levels, and promoting prosocial behaviors such as empathy and cooperation.

Individuals with albinism often face psychosocial difficulties, including stigmatization, social isolation, and increased anxiety, factors that can affect the development and maintenance of interpersonal relationships. In this context, oxytocin may play an important role in improving social adaptation and reducing perceived stress. Evidence from the literature, such as studies by Jin et al. (2023), suggests that the administration of oxytocin can facilitate social interactions and reduce anxiety in vulnerable groups, supporting the idea that this neuropeptide could have a beneficial effect on individuals with albinism who face challenges related to social integration [26].

Recent studies on albinism and its social and psychological impact create the opportunity to investigate the role of oxytocin in this context. Oxytocin is known for its role in facilitating positive social behaviors, including increasing trust and empathy. In the case of individuals with albinism, who can face social ostracism and the psychological impact of stigmatization, investigating the effects and how this hormone can influence social adaptability and emotional resilience becomes essential [27,28]. By examining the interaction between oxytocin and the psychosocial response of individuals with albinism, research can contribute to the development of intervention strategies aimed at improving the quality of life for these vulnerable individuals. Furthermore, a deeper understanding of how oxytocin influences social perception and adaptive behaviors could have practical applications in managing and alleviating the negative effects of social and psychological discrimination associated with albinism [29].

The zebrafish (*Danio rerio*) is considered an ideal research model for the study of albinism for several fundamental reasons. Firstly, zebrafish exhibit significant genetic similarity to humans. Many of the genes that control pigment development and melanocyte function in humans and mice are conserved in zebrafish as well as in other chordates [30,31,32]. Some examples of conserved melanocyte genes that control the analogous cell type, melanophores, in zebrafish, include the transcription factor associated with microphthalmia (referred to as *MITF* in humans, *Mitf* in mice, and *mitf* in zebrafish), dopachrome tautomerase (*DCT*), tyrosinase (*TYR*), tyrosinase-related protein 1 (*TYRP1*), and oculocutaneous albinism 2 (*OCA2*) [33]. In addition to this aspect, zebrafish learn voluntary behaviors through operant conditioning when the same stimuli serve as consequences of behavior. Albino zebrafish models exhibit more pronounced behavioral responses to social and environmental stimuli [34]. Additionally, the lack of pigmentation facilitates detailed analysis of brain structures and neural pathways using imaging techniques, providing an in-depth perspective on the effects of oxytocin on neural networks involved in anxiety and social behavior. Thus, zebrafish have achieved recognition in the neuroscience area and have a high potential to serve as a connecting bridge between biomedical and behavioral sciences, including the study of social behavior [35,36]. Using modern technology, it is possible to monitor the behavioral responses of albino zebrafish treated with oxytocin in various stimulation contexts.

Some studies, such as those presented at the Virtual Reality Symposium of the International Ethology Congress held in 2015 in Cairns, Australia, ref. [37], use robotic fish in the context of anti-predator behavior in zebrafish [38], ultimately addressing the construct validity of paradigms involving zebrafish as biological models of human brain function [39]. Zebrafish have innate abilities to respond to various stimuli, they can hear [13], smell [40], and perceive low-frequency vibrations through their lateral line (structure equivalent to the tactile stimulus perception in terrestrial species). In the present study, we focused on visual stimuli.

In this study, we aimed to investigate the effects of oxytocin treatment on social behavior in albino zebrafish, considering their visual impairments and socio-emotional deficiencies, by using real and virtual stimuli to test social preferences, anxiety levels, adaptability, and responses to risk exposure.

## 2. Results

### 2.1. Behavioral Response of Zebrafish to Predator Video Stimuli—Oxytocin Influences Defensive and Escape Responses in Albino and Non-Albino Zebrafish

Our study investigated whether zebrafish can distinguish between a video stimulus and a real stimulus, considering their reality recognition abilities suggested by the previous literature [41,42]. Our hypothesis was that, if the fish perceived the video stimulus as a real predator, they would exhibit significant changes in behavioral parameters, such as adjusting their distance from the stimulus, freezing duration, turning angle, and swimming velocity. However, if they recognized it as a screen, these variables would remain relatively stable after the initial acclimatization period. To test this hypothesis, the aquarium was divided into two zones: the “screen zone” (where the video stimulus was displayed) and the “safe zone” (a refuge area). The fish were exposed to a video depicting a natural predator (African leaf fish), and behavioral parameters were analyzed at regular intervals. The results indicate significant differences in behavioral responses depending on zebrafish phenotype and the timing of oxytocin administration, supporting the idea that this neuropeptide can influence defensive reactions, but with variable effects depending on the individual characteristics of the fish.

**Figure 1:**
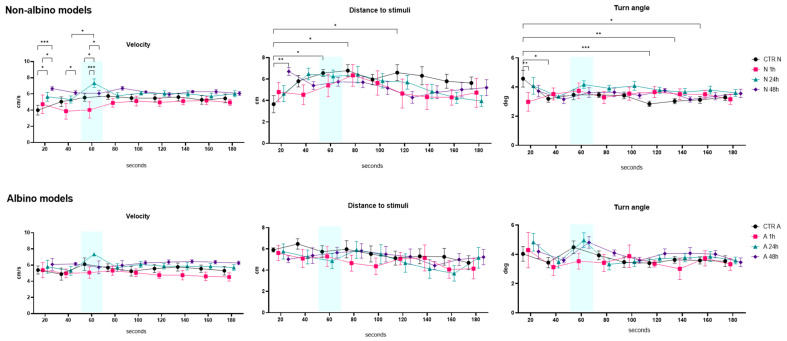
Graphical representation of the velocity (cm/s), distance to stimuli (cm), and turn angle (deg) results for both albino (A) and non-albino (N) zebrafish models in the predator video stimuli test. “1 h/24 h/48 h” represents the exposure time to oxytocin. The blue mark represents the moment when the stimulus appeared during the test. The data are expressed as the mean ± SEM, and a *p* < 0.05 was considered to be statistically significant (* *p* < 0.05; ** *p* < 0.01; *** *p* < 0.001).

**Figure 2:**
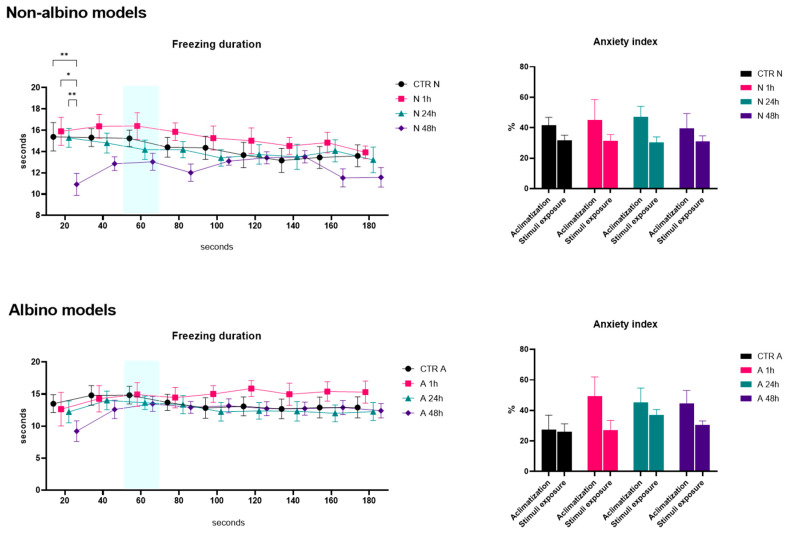
Graphical representation of the freezing duration (seconds) and anxiety index (%) results for both albino (A) and non-albino (N) zebrafish models in the predator video stimuli test. “1 h/24 h/48 h” represents the exposure time to oxytocin. The blue mark represents the moment when the stimulus appeared during the test. The data are expressed as the mean ± SEM, and a *p* < 0.05 was considered to be statistically significant (* *p* < 0.05; ** *p* < 0.01).

In the predator video stimulus test, we obtained several interesting findings. As presented in Figure 1, no significant differences were observed between the albino and non-albino groups in terms of velocity. However, in the non-albino group, significant differences were noted between the groups treated 1 h (N1h) and 48 h (N48h) after oxytocin administration. Specifically, differences were observed in the first 20 s (*p* = 0.048) and at the 60 s interval (when the stimulus appeared) (*p* = 0.031), suggesting that the behavioral response to oxytocin varies over time. For the distance to the stimulus, no significant differences were observed in albino fish, while, in non-albino fish, the control group (CTR N) showed a significant increase in this parameter between 20 and 60 s (*p* = 0.044) and between 20 and 80 s (*p* = 0.019), as well as for 120 s, suggesting the fact that there is a possibility of recognizing the visual stimulus as a real one. Additionally, a significant difference was observed between the CTR N and N48h groups during the first 20 s (*p* = 0.004), indicating that oxytocin treatment at 48 h influences approach behavior toward the stimulus, with higher values in the N48h group compared to the CTR N group. Regarding the turn angle, only non-albino fish displayed differences between the CTR N and N1h groups during the first 20 s (*p* = 0.009), with a lower angle in the N1h group compared to CTR N, and between 20 and 40 s in the CTR N group (*p* = 0.028), suggesting rapid behavioral adaptation to the predator stimulus. As shown in Figure 2, for freezing duration, only non-albino fish exhibited significant changes. A notable difference was observed between the CTR N and N48h groups at 20 s (*p* = 0.004), with a longer freezing duration in the N48h group compared to CTR N, and between the N1h and N48h groups (*p* = 0.01), as well as between the N24h and N48h groups (*p* = 0.005), indicating that the effect of oxytocin on freezing behavior increases significantly, with higher values as time passes after administration. Finally, while the anxiety index did not show significant differences between groups, visual observations suggested a possible anxiogenic effect of oxytocin in albino fish, particularly one hour after administration, when anxiety values were higher in the N1h group compared to CTR N.

Freezing behavior, observed in the predator stimulus test, is clearly defined as an anxiety-related response. It is a defensive behavior specifically associated with fear and anxiety reactions when facing a potentially threatening stimulus. The results showed that, in non-albino fish, oxytocin treatments significantly influenced the duration of freezing, indicating a modification of the defensive response depending on the timing of oxytocin administration (*p* = 0.004 at 20 s and *p* = 0.005 between the groups treated at 24 h and 48 h). This behavior is linked to anxiety levels, and the observed changes suggest that oxytocin may influence this anxiety-related response, particularly when the administration of oxytocin is longer.

Our findings show that non-albino zebrafish exhibit significant behavioral responses to a predator video, while albino zebrafish do not. Oxytocin influenced defensive behavior in non-albino fish, especially 48 h post-administration, affecting distance to the stimulus (*p* = 0.004), turning angle (*p* = 0.009), and freezing duration (*p* = 0.004). These findings suggest that zebrafish can recognize virtual stimuli, with oxytocin modulating their responses based on phenotype and administration timing.

### 2.2. Influence of Video vs. Real Conspecifics on Zebrafish Behavior—Oxytocin Affects Anxiety and Social Behaviors in Albino and Non-Albino Zebrafish

Our hypothesis was that zebrafish can distinguish between a video stimulus and a real fish, and that oxytocin can influence this ability. Therefore, we aimed to test their preference between a real conspecific and a virtual one. If the fish perceive the video as a real conspecific, we expect them to spend a similar amount of time near the screen and the live fish. If they recognize the difference, they should prefer interacting with the real fish. To test this hypothesis, we used the conspecific video vs. real fish preference test, comparing the behavior of albino and non-albino zebrafish before and after oxytocin administration. To test this, we used a simple maze setup, with a two-option environment: a screen displaying a zebrafish video and a compartment containing a real fish. Fish activity was recorded by an overhead camera. Each fish was placed individually at the center of the arena and allowed to explore freely, choosing between the two options.

**Figure 3:**
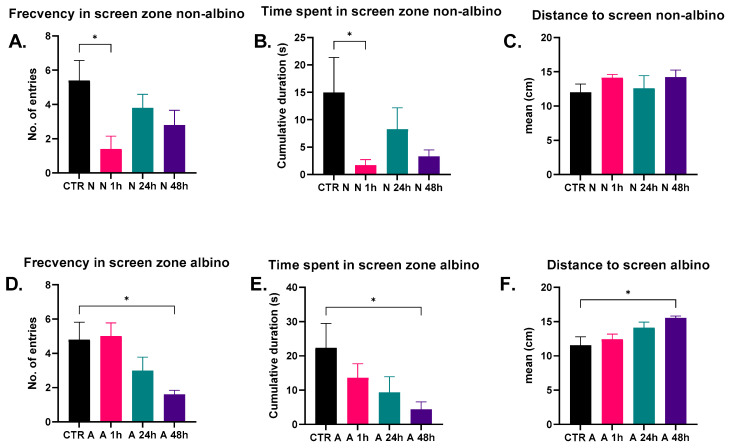
Graphical representation of the results for both albino and non-albino zebrafish models in the conspecific video vs. real fish preference test ((**A**) Frecvency in screen zone non-albino; (**B**) Time spent in screen zone non-albino; (**C**) Distance to screen non-albino; (**D**) Frecvency in screen zone albino; (**E**) Time spent in screen zone albino; (**F**) Distance to screen albino). The data are expressed as the mean ± SEM, and a *p* < 0.05 was considered to be statistically significant (* *p* < 0.05).

In the preference test between the video stimuli and real fish, the behaviors of albino and non-albino zebrafish were evaluated regarding the time spent in the screen vicinity, frequency of interactions with it, and distance from the screen. Statistical analysis revealed several significant results suggesting the influence of oxytocin on the social behavior of these models (Figure 3). The time spent in the screen zone was significantly higher in the group treated with oxytocin for 48 h (A48h) compared to the albino control group (CTR A) (*p* = 0.048). Similarly, in the non-albino fish, the time spent in the screen zone was significantly higher in the group treated with oxytocin for 1 h (N1h) compared to the non-albino control group (CTR N) (*p* = 0.048). This suggests an effect of oxytocin on social behavior. The frequency of interactions with the screen was significantly higher in the albino group treated with oxytocin for 48 h (A48h) compared to the albino control group (CTR A) (*p* = 0.023). Similarly, in non-albino fish, the frequency of interactions was significantly higher in the group treated with oxytocin for 1 h (N1h) compared to the control group (*p* = 0.018), indicating a positive effect of oxytocin on social interactions. In albino fish, the distance from the screen was significantly smaller in the group treated with oxytocin for 48 h (A48h) compared to the control group (CTR A) (*p* = 0.011), suggesting a closer approach to the visual stimulus. In contrast, no significant differences in distance from the screen were observed in non-albino fish.

In short, in albino zebrafish, the time spent near the screen and the number of interactions significantly increased after 48 h of oxytocin treatment. In non-albino zebrafish, oxytocin administered for 1 h also increased the number of interactions, but there were no significant changes in the distance from the screen. These results suggest that oxytocin affects social behavior, with the effects varying depending on the zebrafish phenotype (albino or non-albino) and the timing of administration.

## 3. Discussion

According to research conducted by Onarheim and colleagues (2022), anxiety behaviors in zebrafish are characterized by parameters such as freezing duration, reaction speed to the predator stimulus, and the anxiety index, which reflects defensive responses and high stress levels. These behaviors are associated with avoiding open areas and reduced activity in light conditions, indicating a state of distress [43]. In contrast, social behaviors are expressed through time spent near conspecifics, the frequency of social interactions, and the distance from the screen—factors that reflect the desire for social affiliation and exploratory behaviors. According to the research by Fontana et al. (2021), social behaviors are related to interactions between individuals and the desire to approach conspecifics, being correlated with increased social activity, especially in a stimulating environment [44].

The previous research complemented by our study provides a comprehensive understanding of the effects of oxytocin on social behavior and anxiety in zebrafish, highlighting both similarities and differences between various phenotypes and the duration of the treatment. Existing studies suggest that oxytocin can influence social and anxiety-related behaviors in zebrafish, with its effects being dependent on the social context and type of treatment administered [45]. As suggested by studies in the literature, Ricci et al. (2013) indicate that oxytocin receptors may respond differently depending on the fish’s age or social context, and, in our study, significant differences were observed between albino and non-albino fish [46]. Additionally, Chuang et al. (2021) suggest that oxytocin may modulate not only social behaviors but also defensive reactions, being more effective in reducing anxiety and behaviors related to speed and freezing, particularly in non-albino fish [47].

The anxiogenic effect observed in the group treated with oxytocin at 48 h could be explained by the influence of oxytocin on the hypothalamic–pituitary–adrenal (HPA) axis. Although oxytocin is well-known for its anxiolytic effects, previous studies suggest that it may have bidirectional effects on emotional stress, depending on the context and individual factors [48,49]. Specifically, oxytocin can amplify stress responses when individuals are exposed to unfamiliar environments or threatening stimuli [46,47]. This hypothesis is supported by studies showing that oxytocin administration can increase amygdala reactivity to negative stimuli, potentially leading to a paradoxical increase in anxiety under certain conditions [50,51]. Another possible mechanism for the observed anxiogenic effect could be the influence of oxytocin on amygdala activity [52]. While oxytocin may reduce amygdala activity in positive social contexts, some studies have shown that it can have the opposite effect in stressful situations, increasing neuronal reactivity and amplifying defensive responses. The study by Labuschagne et al. (2010) explores the effects of oxytocin on amygdala reactivity in stressful contexts, highlighting how this neuropeptide can influence emotional responses [52]. This reaction may explain why, in our predator stimulus test, the oxytocin-treated fish exhibited a longer freezing behavior, indicating an increase in anxiety in the presence of a threatening stimulus.

Regarding anxiety-like behaviors and defensive responses, our research highlighted an anxiogenic effect of oxytocin, particularly in the group treated for 48 h. This group exhibited a longer duration of freezing, suggesting a suppression of defensive reactions, an effect also observed in previous studies. For example, Gemmer et al. [53] and evidence from other studies suggest that oxytocin regulates aggression and defensive behaviors in zebrafish. In our study, long exposure time effects were more pronounced in the non-albino fish groups. Thus, it is confirmed that oxytocin can have a significant impact on defensive behavior and anxiety, but its effects may vary depending on phenotype and treatment duration.

Furthermore, we observed a significant increase in the preference for social interactions in albino fish, especially after 48 h of oxytocin treatment, which aligns with the existing literature suggesting that oxytocin contributes to the development and maintenance of social behavior [54]. Prior studies have shown that exposure to oxytocin activates the social system in zebrafish, with social behaviors being more prominently regulated by oxytocin, particularly in the context of interactions with conspecifics. Social interaction behaviors were clearly associated with social preference rather than anxiety reduction. For example, albino fish treated with oxytocin at 48 h (A48h) showed a significant increase in the frequency of interactions with the screen (*p* = 0.023) and a greater interest in real conspecifics (*p* = 0.011 compared to the albino control group). These results suggest that oxytocin influences social behaviors by enhancing social interaction rather than by reducing anxiety. Thus, we have clarified that oxytocin reduces anxiety (by influencing freezing behavior) and enhances social behaviors (by increasing interaction frequency and interest in real conspecifics). In addition, non-albino fish maintained a consistent behavior towards stimuli, while albino fish became more attracted to real conspecifics after oxytocin treatment. These results suggest that oxytocin influences social behaviors differently, depending on the zebrafish phenotype, contributing more to the social comfort of albino fish. These observations are supported by prior studies, which show that the effects of oxytocin depend on the type of stimuli and social context [55,56].

Prior studies also support our results, such as those of Ricci et al. (2013) on the effect of interdependent movements on the social preferences of zebrafish. Their results showed a significant preference for interacting with socially interdependent stimuli animated in 3D [46]. Similarly, the study by Chuang et al. (2021) explored an innovative system for the simultaneous observation of the behavior of eight adult zebrafish to analyze their responses to these visual stimuli. They found that zebrafish exhibit specific reactions to visual stimuli, including increased exploratory abilities that elicit a negative optomotor reaction [47]. Our results show that the responses to virtual stimuli vary depending on the zebrafish phenotype (albino or non-albino) and the timing of oxytocin administration, suggesting the high level of impact of oxytocin exposure modulations on this aspect. For example, the time spent near the screen zone in the social preference test decreases with increasing duration of oxytocin treatment in albino fish.

Regarding the visual stimuli, Gerlai R. (2017) used, in his study, animated images to examine zebrafish behavior, allowing for the simulation of visual stimuli and observation of their reactions in a controlled environment (e.g., exploration, avoidance reactions, freezing behavior, and social interaction). This method aids in understanding both instinctive and learned behaviors as well as social interactions [37]. In our study, we investigated the relevance of oxytocin, which regulates the behavior and emotional responses of zebrafish in the context of visual and social stimuli.

A connection between oxytocin and the dopaminergic system could be observed, which regulates social behaviors and emotional processes. Singh and Ousby (2021) demonstrated that oxytocin modulates the social preferences of zebrafish, with its effects varying based on their phenotype—a finding confirmed also in our research, where albino fish exhibited a positive adaptation to social stimuli compared to non-albino fish [57]. Moreover, the study by Umeoka et al. (2020) deepens the understanding of the interaction between oxytocin and the dopaminergic system, indicating that oxytocin influences dopamine release in the nucleus accumbent, having a differential impact on social behavior and direct interactions. This observation is in line with our results, where oxytocin had a more significant effect on social behavior in albino zebrafish; this could suggest a direct link between the dopaminergic system and social responses to stimuli [58]. Additionally, Silva et al. (2019) outlined the anxiolytic effects of oxytocin in zebrafish models by exploring the role of oxytocin receptor subtypes. These findings, along with ours, which showed a reduction in anxiety-like behavior and defensive responses in oxytocin-treated groups, indicate that oxytocin not only regulates social behaviors but also emotional and defensive responses [59].

Additional knowledge is provided within our findings in the field of confirming that oxytocin plays an important role in the modulation of social behavior and emotional responses in zebrafish, particularly with regard to social interactions and anxiety behaviors. Consistent with the existing evidence that oxytocin promotes positive social behaviors and reduces aggressive and defensive behaviors, albino fish benefited more from oxytocin in terms of social comfort and desire to interact with conspecifics [60].

Our study suggests a potential role of oxytocin in improving social interactions in albino zebrafish models, adding knowledge to the evidence that many of the fundamental mechanisms of oxytocin are evolutionarily conserved, making these results relevant for further research in mammals and humans, especially regarding the time of oxytocin administration. Studies in mice and primates have shown that oxytocin influences social behaviors and reduces stress, while, in humans, oxytocin administration has been associated with improved social interactions [61,62]. However, further research is needed, and this study’s limitations should be considered to validate these results in more complex contexts.

## 4. Materials and Methods

### 4.1. Ethical Note

All animals were treated and maintained in accordance with the EU Commission Recommendation (2007), Directive 2010/63/EU of the European Parliament, and the Council of 22 September 2010 guidelines for the accommodation, care, and protection of animals used for experimental and other scientific purposes. The protocol we followed received approval from the Ethics Committee of the Faculty of Biology, “Alexandru Ioan Cuza” University, Iasi, with registration no. 1349/20 March 2024.

### 4.2. Animal Maintenance

For our study, we used 70 adult zebrafish (*Danio rerio*), 35 normal fish (non-albino) (N), and 35 albino fish (A) from an authorized breeder. The zebrafish used in this experiment had an acclimatization period under experimental laboratory conditions for three weeks prior to the experiments in 10 L aquariums equipped with oxygen pumps and with the water changed daily. After this period, the zebrafish were randomly distributed into experimental groups (n = 10/5).

### 4.3. Experimental Design

The zebrafish were randomly selected and acclimatized within the corresponding groups under the conditions mentioned above. We created eight experimental groups to be exposed to oxytocin treatment at a concentration of 33.2 ng/mL for 15 min. According to previous studies, oxytocin remains active at room temperature in water for 21 days with normal function. Oxytocin was purchased in pharmaceutical liquid form (Pasteur Company, Bucharest, Romania, 10 I.U./mL). The dose of oxytocin was determined in accordance with the existing literature [63,64,65]. Behavioral responses were analyzed 24 h after the first dose (groups A 24 h, N 24 h) (n = 10) and 48 h after the administration of two doses (groups A 48 h, N 48 h) (n = 10), along with two control groups for each specific model (CTR A, CTR N) (Table 1). In addition, two groups with n = 5 received the same experimental dose for 15 min and were tested 1 h after exposure (A 1 h, N 1 h). The following test was assessed:

#### 4.3.1. Predator Video Stimulus Test

**Figure 4:**
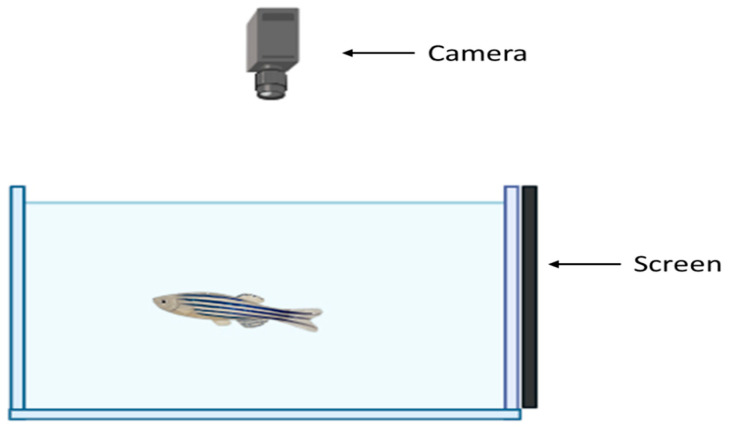
Experimental setup of the predator video stimulus test. Image created in Biorender.com www.biorender.com Accessed in 2 April 2024.

The experimental apparatus consisted of a rectangular tank measuring 30 cm × 20 cm × 20 cm, filled with 6 L of water, equipped with a camera above to record the fish’s activity during the test. A monitor covering the entire right side of the tank was placed on the right side (Figure 4). The fish were placed in the apparatus for a 1 min acclimatization period without visual stimuli, after which a video featuring an African leaf fish (its natural predator [66]) was played for 2 min. The distance to the visual stimuli, speed, turning angle, and freezing behavior were recorded using the EthoVision XT 16 Software, Noldus, Netherlands, every 20 s as the mean of the sample once every 0.4 s.

Our hypothesis, based on previous observations made by us, was that the distance to the visual stimuli, freezing, turning angle, and velocity (due to high-speed escape episodes) would increase upon the presentation of stimuli if they recognized it as a real predator; otherwise, all these parameters would remain normal without specific variations every 10 s, especially after the 1 min acclimatization period [67].

In addition, we evaluated the anxiety index based on suggestions from the literature and previous studies for both acclimatization and stimulus exposure periods. The anxiety index was calculated as the time spent in the peripheral zone (peripheral areas) divided by the total testing time (60 s for acclimatization and 120 s for stimulus exposure) multiplied by 100 [68,69].

#### 4.3.2. Video Conspecific vs. Real Fish Preference Test

**Figure 5:**
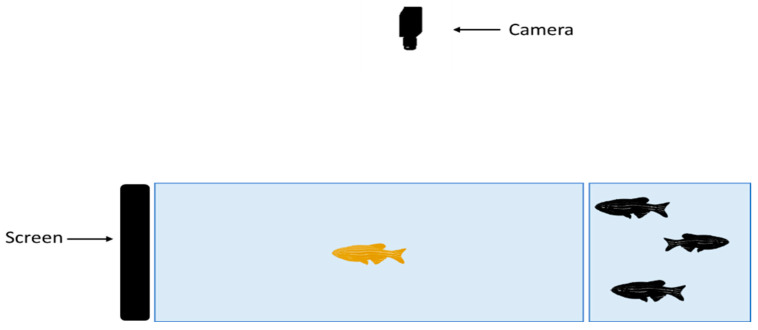
Experimental setup for the video conspecific vs. real fish preference test. Image created in Biorender.com www.biorender.com Accessed in 2 April 2024.

The experimental configuration was carried out using a cross maze, closing one arm with lids. The testing arena consisted of a rectangular tank measuring 20 cm × 10 cm × 10 cm that was made up of two arms which were filled with water and equipped with a camera above to record the fish’s activity. In the left arm, a monitor displayed a video stimulus featuring a zebrafish (Figure 5); for the albino models, we used a video of the same fish, and vice versa. A real zebrafish was placed in the right arm. To initiate the test, the fish were placed one by one in the center of the testing arena and allowed to explore the environment, reacting quickly and concisely while choosing between the options. The time spent in the video stimulus zone and the number of entries into it were recorded by the EthoVision XT 16 Software, Noldus, Netherlands, for 2 min. Additionally, the distance to the visual stimuli was recorded every 20 s as the mean of the sample once every 0.1 s.

### 4.4. Statistical Analysis

The normality and distribution of the data were determined by the Shapiro–Wilk test using the Graph Pad Prism 9 software (San Diego, CA, USA). Outliers were removed by ROUT analysis. Multiple comparisons between the groups (by time and group) and post hoc analysis were then performed using Two-way ANOVA followed by Šídák’s multiple comparisons test for the predator video stimuli test and One-way ANOVA followed by Dunnet’s multiple comparison test for the conspecific video vs. real fish preference test. The data are expressed as the mean ± SEM, and a *p* < 0.05 was considered to be statistically significant.

## 5. Conclusions

Our study suggests that oxytocin plays an important role in enhancing social interactions in albino zebrafish, indicating a potential neurochemical mechanism that may also be relevant to other species, including mammals. Additionally, our findings highlight the zebrafish’s ability to recognize and differentiate between real and virtual stimuli, suggesting a stimulus discrimination process that can be influenced by oxytocin administration. However, it is important to note that extrapolating these results to higher species, such as mammals or humans, should be performed cautiously, given the complexity of their neurobiological systems. Additionally, clinical studies investigating the effects of oxytocin on social behavior in humans have shown mixed results, indicating that therapeutic interventions with oxytocin need further studies and should be approached with caution and personalized according to each patient’s needs.

Based on our findings, we conclude that the preference test between video conspecifics and real fish revealed that oxytocin enhances social behaviors in zebrafish, particularly in albino models. Albino fish showed increased interactions and spent more time near real conspecifics, especially 48 h after oxytocin treatment, indicating that oxytocin promotes social engagement and reduces social anxiety. In contrast, non-albino fish exhibited stable interactions with both stimuli, suggesting that oxytocin’s effects are more pronounced in fish with sensory or social deficits, such as albinos. This highlights the potential of oxytocin in improving social cognition and adaptation.

Additionally, the conspecific video vs. real fish preference test showed that oxytocin’s impact on predator stimulus responses varied in non-albino fish, depending on the time elapsed since administration. While albino fish did not show significant differences in most parameters, the effects observed in non-albino groups suggest that oxytocin plays a role in regulating defensive and anxiety-related behaviors. These findings underscore the need for further research on oxytocin’s influence on social interactions and threat responses both in albino and non-albino zebrafish models.

Regarding the theoretical foundation, previous research on mammals and humans has demonstrated that oxytocin plays a role in forming and maintaining social bonds, as well as in cognitive processes related to recognizing and interpreting social signals. However, the existing literature also highlights limitations in using oxytocin for therapeutic purposes, such as the variable effects between individuals and the risks associated with its irregular or high-dose administration. Therefore, while this study provides a promising starting point for future research, it is essential to consider these limitations and continue investigating the impact of oxytocin on social behaviors in a clinical context. Future studies should ideally employ more rigorous approaches and closely assess how different oxytocin levels influence social and therapeutic behaviors in individuals with social behavior disorders.

## Figures and Tables

**Table 1:** Experimental group assignment.

Experimental Group	No. of Individuals	Oxytocin Concentration	Time of Exposure (hours)
CN	10	33.2 ng/mL	-
CA	10	33.2 ng/mL	-
A24h	10	33.2 ng/mL	24
N24h	10	33.2 ng/mL	24
A48h	10	33.2 ng/mL	48
N48h	10	33.2 ng/mL	48
A1h	5	33.2 ng/mL	1
N1h	5	33.2 ng/mL	1

## Data Availability

Data is contained within the article.
